# Evaluation of New Hollow Sleeve Composites for Direct Post-Core Construction

**DOI:** 10.3390/ma14237397

**Published:** 2021-12-02

**Authors:** Shinji Yoshii, Sufyan Garoushi, Chiaki Kitamura, Pekka K. Vallittu, Lippo V. Lassila

**Affiliations:** 1 Department of Biomaterials Science and Turku Clinical Biomaterial Center—TCBC, Institute of Dentistry, University of Turku, 20500 Turku, Finland; sufgar@utu.fi (S.G.); pekval@utu.fi (P.K.V.); liplas@utu.fi (L.V.L.); 2 Division of Promoting Learning Design Education, Kyushu Dental University, Fukuoka 803-8580, Japan; 3 Division of Endodontics and Restorative Dentistry, Kyushu Dental University, Fukuoka 803-8580, Japan; r06kitamura@fa.kyu-dent.ac.jp; 4 City of Turku Welfare Division, Oral Health Care, 20101 Turku, Finland

**Keywords:** fiber post, core construction, hollow fiber, flexural properties

## Abstract

The preset shape and diameter of a prefabricated FRC post rarely follows the anatomy of the root canal. To solve this problem, a new hollow sleeve composite (HSC) system for post-core construction was developed and characterized. A woven fiber was impregnated with two types of resins: Bis-GMA or PMMA, and rolled into cylinders with outer diameter of 2 mm and two different inner diameters, namely 1.2 or 1.5 mm. The commercial i-TFC system was used as a control. Dual-cure resin composite was injected into these sleeves. Additionally, conventional solid fiber post was used as the inner part of the sleeve. The three-point bending test was used to measure the mechanical properties of the specimens and the fracture surface was examined using an electron microscope (SEM). The HSC (1.5 mm, Bis-GMA) revealed a statistically similar flexural modulus but higher flexural strength (437 MPa) compared to i-TFC (239 MPa; ANOVA, *p* < 0.05). When a fiber post was added inside, all values had a tendency to increase. After hydrothermal accelerated aging, the majority of specimens showed a significant (*p* < 0.05) decrease in flexural strength and modulus. SEM fracture analysis confirmed that the delamination occurred at the interface between the outer and inner materials. The HSC system provided flexibility but still high mechanical values compared to the commercial system. Thus, this system might offer an alternative practical option for direct post-core construction.

## 1. Introduction

Endodontically treated teeth (ETT) with a significant coronal loss of dentin may need additional support to retain coronal restoration. Nowadays, the use of prefabricated fiber-reinforced composite (FRC) posts is recommended mainly in cases of severe loss of remaining coronal tooth structure to promote the retention of the final restoration and to optimize the biomechanical behavior of the remaining tooth structure [1,2]. There is wide variation between FRC posts in respect to fiber types, fiber orientations, fiber surface treatments, and methods of incorporating the fibers into the resin matrix [3]. Glass FRC posts are the most widely used reinforcing fibers with high tensile strength and good bonding to the resin matrix [4]. Despite the favorable biomechanical behavior of prefabricated glass FRC posts, there has been discussion about the shortcomings as well [2,5,6]. The preset shape and diameter of a prefabricated FRC post rarely follows the anatomy of the root canal. Consequently, when inserting a prefabricated FRC post, a big space will be filled with resin cement coronally and a needless amount of dentin may have to be removed apically. Additionally, the inadequate bonding ability of a cross-linked prefabricated FRC post to resin cements, composite core materials, and root canal dentine is emphasized [7].

With time, a poor bonding between an FRC post and resin cement may result in marginal breakdown and further secondary caries. Thus, some researchers consider that conventional post-core systems especially for large and flared root canals are an inefficient construction method [2,5,7,8]. To strengthen large root canals, additional auxiliary fiber posts were used to support the primary fiber post [9,10]. Keeping in mind that the endodontic canal is often a flared form and even if auxiliary posts are used, the problem of resin space in the cervical region cannot be completely solved. However, the best way to restore ETT has long been and still is a subject of debate.

The i-TFC system (Sun Medical Co., Ltd., Moriyama, Japan) is one of the newly developed fiber post systems for wide and flared root canal. This system consists of a fiber post and a fiber hollow sleeve [11,12,13]. When fiber hollow sleeve composite (HSC) system for post-core construction is developed, fiber orientation and design are needed to be carefully studied.

The i-TFC fiber sleeve system uses a 20-degree braided fiber structure. Fiber orientation (direction) influences the mechanical properties and reinforcing efficiency (Krenchel’s factor) of the FRC structure [14,15]. Continuous unidirectional fibers, such as those in most prefabricated posts, provide strength, stiffness, and anisotropic mechanical strength to the composite in the direction of the fibers, thus they are suitable for applications in which the direction of the stresses is known. Furthermore, continuous bidirectional (woven) fibers have reinforcing fibers in two directions, thus they have a reinforcing effect equally in two directions [14,15].

Taking into account the positive effect of woven fiber reinforcement and negative side effects of prefabricated unidirectional FRC posts, the question arises: could we have another fiber reinforcement option for post-core construction to restore ETT teeth? To our best knowledge, very little research exists in this field. Therefore, the goal of the current research study was to develop a novel HSC system using woven fiber sheets, which can be molded into any shape, and to investigate its mechanical performance in comparison with the commercial i-TFC system. Furthermore, the impact of different impregnation monomers, diameters, and hydrothermal accelerated aging and filling/inner materials on the mechanical performance of the experimental HSC was evaluated.

## 2. Materials and Methods 

Table 1 lists the materials used as well as information on their manufacturing and composition.

### 2.1. Preparation of HSC System

**Figure 1:**
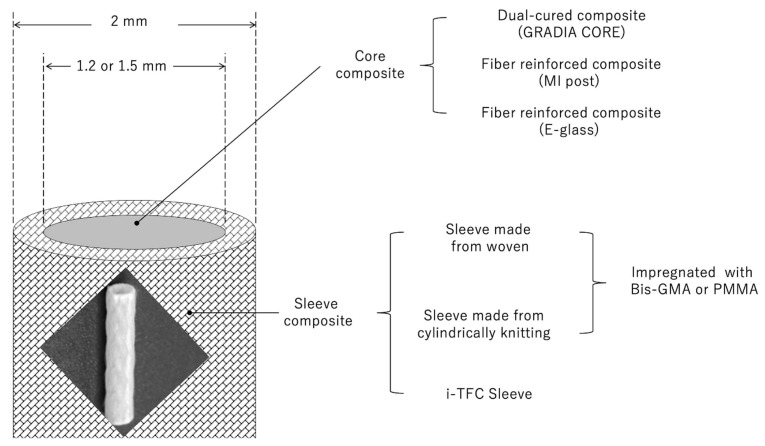
Schematic drawing of hollow sleeve composite.

Very thin glass fiber weaves or cylindrical braided fibers were impregnated with either Bis-GMA/TEGDMA monomers to form the hollow sleeve. Two different diameters (1.2 mm and 1.5 mm) of stainless-steel rods were used as a mold. Glass fibers were wrapped around rods (16 mm in length) to form hollow sleeves with an outer diameter of 2 mm. The resin matrix was light-cured by a hand-light curing device for 20 s (Elipar S10, 3M ESPE, St. Paul, MN, USA) with an irradiance of 1200 mW/cm^2^. After the polymerization of the fiber sleeve, the hollow sleeve was filled with different composites (Figure 1). Gradia Core was injected and cured into the hollow space. The MI post was inserted into the hollow space and bonded with Gradia Core. E-glass was inserted into the hollow space and bonded with Bis-GMA. All prepared bars were additionally polymerized for 20 s using a hand-light curing device.

As a control, the commercial i-TFC glass system was used with the same outer diameter of 2 mm. The i-TFC system consisted of a hollow sleeve filled with a 1.5 mm i-TFC fiber post. We inserted the fiber post into the i-TFC sleeve with i-TFC resin cement, followed by light curing for 20 s. A total of 108 specimens were prepared (*n* = 12/group) and divided into 9 groups (Table 2). Specimens per each group were then either dry stored in a desiccator (room temperature) for 24 h (*n* = 6/group) or aged before testing (*n* = 6/group). The specimens were aged in boiling deionized distilled water for 16 h. In order to do this, the specimens were placed in glass bottles of 40 mL distilled water and their caps were tightened. The bottles were then set in a heating oven for 16 h at 100 °C. Table 2 shows all fabricated cylindrical bar groups and study designs.

### 2.2. Flexural Strength and Modulus of Elasticity

The three-point bending test was used to measure the mechanical properties of the specimens adapted to the ISO 10477 standard (span, 10 mm; crosshead speed, 1.0 mm/min; cross-sectional diameter of loading tip, 2 mm) [16]. All specimens were tested with a material testing machine (modelLRX, Lloyd Instruments Ltd., Fareham, UK) and the stress–strain curves were recorded with a PC-computer program (Nexygen, Lloyd Instruments Ltd., Fareham, UK) using a speed of 1.0 mm/min on a 10 mm span. The outer diameter of each specimen was measured before testing.

### 2.3. Microscopic Analysis

After the mechanical tests, FRC bars were cut with cutting stick at the part of the broken cross-section and embedded into the acrylic resin. It was polished to #1500 and then the cross-sections of the specimens were gold coated (BAL-TEC SCD 050 Sputter Coater, Balzers, Liechtenstein) for examination using a scanning electron microscope (SEM; JSM-5500, JEOL Ltd., Tokyo, Japan). SEM analysis was performed at an operating voltage of 15 kV, spot-size of 37, and working distance of 18 mm. SEM photomicrographs were used for visual analysis of the fiber geometry and layer adaptation. In addition, FRC bars were examined by SEM perpendicular to the sleeve surface for measuring the fiber roving’s angulation to the long axis of the bar.

### 2.4. Statistical Analysis

The resulting data were analyzed statistically using a multi-way ANOVA test, followed by one-way ANOVA and Tukey’s HSD test (α = 0.05) to illustrate the differences between the groups by SPSS version 23 (SPSS, IBM Corp., New York, NY, USA).

## 3. Results and Discussion

We aimed to develop a new fiber system that fits to any root canal. In this study, we made a new hollow sleeve composite system by ourselves with an outer diameter of 2 mm and length of 16 mm to compare to a commercial system with the same dimensions. This method involves rolling up the woven impregnated fiber into cylinders, which can be molded into various shapes.

**Figure 2:**
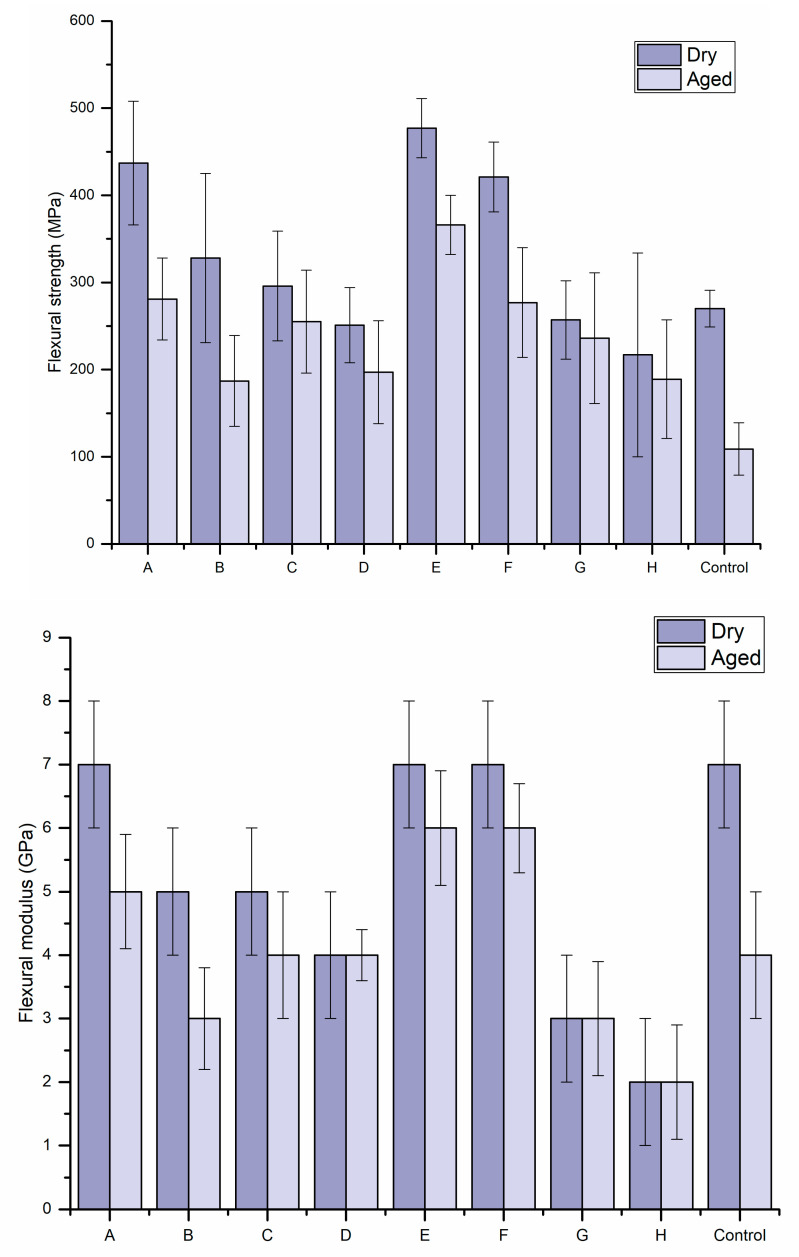
Bar graph showing mean flexural strength (MPa) and flexural modulus (GPa) with the standard deviations (SD) of the tested groups.

The results of the three-point bending test (flexural strength and modulus) of the tested groups are shown in Figure 2. Despite the type of inner/core material, when the inner diameter of the hollow sleeve was 1.5 mm and the sleeve material was woven fiber with Bis-GMA (Groups A, E, and F), the flexural strength in dry conditions was significantly higher than the other groups. Furthermore, the control group in dry conditions showed similar flexural modulus values to groups A, E, and F. Different material combinations revealed considerable variations between values. However, the presence/absence of air bubbles or wetting defects during specimen manufacturing explains the large standard variation in some groups.

**Figure 3:**
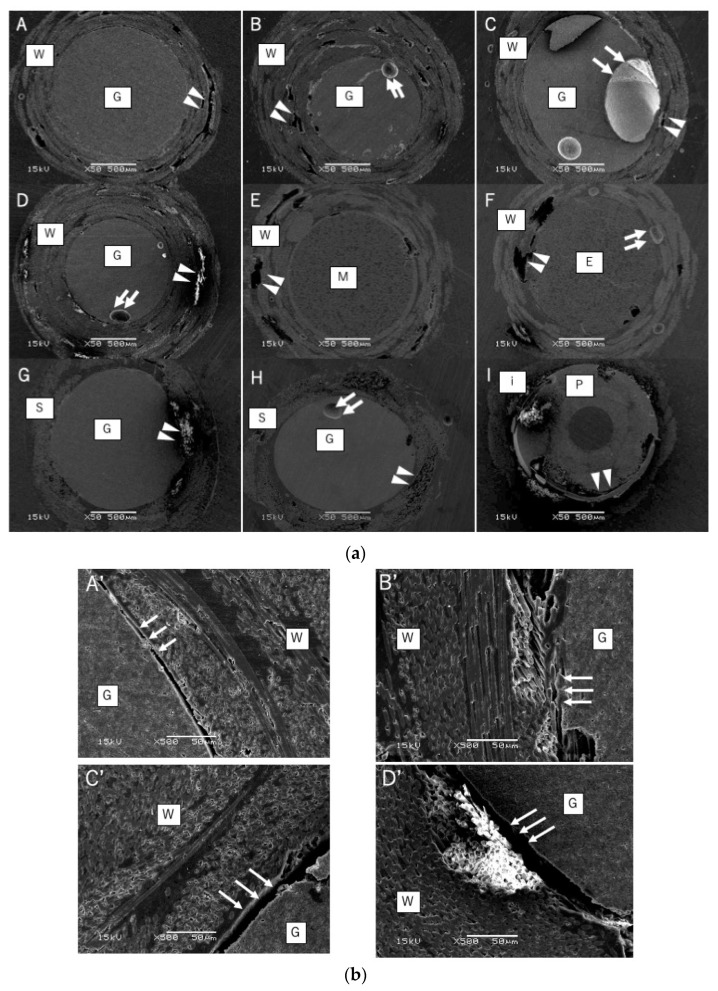
(**a**) SEM images (×50) of the cross-sections of fracture surfaces for various specimens. The alphabet on the upper left of each figure indicates the code of Table 2. W: woven, G: Gradia core, M: MI post, E: E-glass, S: sleeve fiber net, i: i-TFC sleeve, and P: i-TFC post. Arrowheads indicate the crush point, while arrows indicate the void. (**b**) SEM images (×500) of the cross-sections of fracture surfaces for various specimens. Each figure is an enlarged view of the same alphabet in Figure 3a. W: woven and G: Gradia core. Arrows indicate the delamination part between the outer fiber sleeve and the inner core.

In addition, through observation by SEM, Figure 3a shows the signs of crushing on the sleeve at the part of the broken cross-section. In fact, all specimens were fractured inside the sleeve or between the sleeve and the internal material.

Firstly, materials were compared to specimens of the same inner diameters (Groups A and B, C and D). Fibers impregnated with Bis-GMA resin performed better than those impregnated with PMMA (Figure 2). SEM examination of the cross-sections of FRC sleeves showed that delamination occurred between the outer fiber sleeve and the inner core after the test (Figure 3b). In addition, Figure 3b shows that the sleeve fiber was oriented in two directions.

This result suggests that the Bis-GMA resin has better adhesive ability and wettability than PMMA.

Surprisingly, the groups with smaller inner diameters (1.2 mm) provided lower mechanical values than the 1.5 mm groups in all measurements (Groups A and C, B and D). One of the reasons for this is the fact that all values depend on the thickness of the inner materials. When the inner diameter was 1.5 mm and the sleeve material was woven fiber with Bis-GMA, the group had a similar flexural modulus but higher strength than i-TFC.

Cylindrically braided knitted fiber had the lowest flexural modulus due to the complicated knitting pattern of the fiber net, which prevents the resin from penetrating sufficiently.

**Figure 4:**
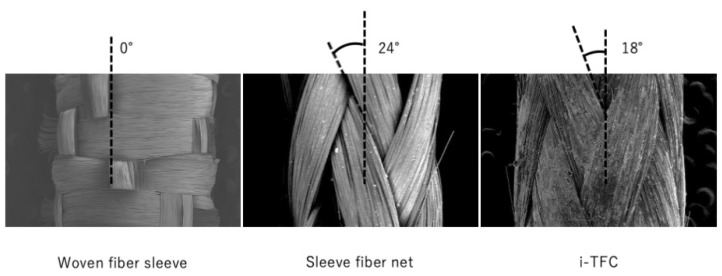
SEM images of the cross-sections of fracture surfaces for various specimens.

SEM showed the fiber roving orientation to be 0 degrees at the woven fiber, 18 degrees at the i-TFC, and 24 degrees at the cylindrically knitted fiber (Figure 4) The fiber roving orientation had an effect on these results because the smaller the axis, the larger the value of the flexural modulus and max-bending stress [17].

In the other series of experiments, where the inner materials were compared, both the E-glass or MI post groups had higher flexural strength values than i-TFC and similar flexural moduli (Groups A, E, F, and I). This result suggests that the bending strength can be changed freely by changing the inner material without changing the elastic modulus.

The contemporary fiber posts available in the market are effective for the treatment of teeth in normal conditions and for most cases. However, in cases where flared root canals exist, conventional fiber post treatment may not be the optimal choice [12,18]. Therefore, there is room for improvement in the construction methods involving fiber posts. We proposed a new hollow sleeve composite system that follows these concepts. In addition, it is possible to adapt these systems to various shapes and sizes.

In this study, we were able to create a system with various physical properties by changing the inner diameter and internal material. They are easy to create and can be used not only for the anterior teeth but also for molars where the available materials may not always be suitable. In contrast, as specimens were hand-impregnated, occasionally, bubbles were observed in the SEM images of all the specimens at the interface joining the outer and inner materials, i.e., the resin and the rolled up woven fiber sheet (Figure 3). A non-polymerized layer remained around the bubbled parts, making the materials mechanically weak and acting as an initiation point for crack. The next step to improve these systems would be to consider a pressurized method to fabricate specimens without air bubbles.

In the current research study, FRC specimens were submitted to artificial hydrothermal accelerated aging by boiling the specimens in water. The length of boiling time was according to previous studies, which reported a decrease in the dental composite strength after the first 16 h of immersing specimens in boiling water [19,20]. As a result, the flexural strength and modulus were significantly decreased after hydrothermal accelerated aging (*p* < 0.05), except for groups D, G, and H which showed no differences in the flexural modulus.

It is important to highlight that in this research study, only a portion of the mechanical performance of hollow sleeve composites were investigated; other clinically important parameters, such as bonding at interfaces, adaptability, crown loading, light transmission, and radiopacity, were not evaluated. Further evaluation is needed to confirm the optimal clinical application.

## 4. Conclusions

In this study, we developed a new HSC system that can be modified for various post cavities. Within the limitations of this study, the following conclusions can be drawn for HSC systems:

1. Sleeve impregnated with Bis-GMA resin mechanically performed better than those impregnated with PMMA;
2. The flexural strength of the HSC system depends on the type of inner material;
3. The mechanical properties of the HSC system can be modified by changing the inner diameter of the sleeve composite;
4. Hydrothermal accelerated aging decreased the flexural properties compared to the control condition for the majority of the tested HSC groups; and
5. Fiber orientation has an essential effect on the mechanical performance of the HSC system.

## Figures and Tables

**Table 1:** Materials used in this study.

Material	Manufacturer	Lot.	Composition
Woven fiber sheet	HEXCEL, Stamford, CT, USA	85M208171309z	E-glass
Cylindrically knitted fiber	Siltex, Julbach, Germany	582	E-glass
i-TFC sleeve	Sun Medical Co., Ltd., Moriyama, Japan	SK1	E-glass and UDMA-based matrix resin
i-TFC sleeve	Sun Medical Co., Ltd., Moriyama, Japan	SK1	E-glass and UDMA-based matrix resin
Gradia Core	GC Europe, Leuven, Belgium	1804161	Alumino-silicate glass and silicon dioxide (70 wt%); UDMA and other dimethacrylate (30 wt%)
MI Core Fiber Post	GC Corp, Tokyo, Japan	20170728	UDMA, PMMA, and glass fibers
E-glass fiber raw	Owens Cornig, Houston, TX, USA	160805	E-glass

UDMA, urethane dimethacrylate; PMMA, polymethylmethacrylate; wt%, weight percentage.

**Table 2:** Designed groups for the three-point bending test (n = 6/group).

Code	Sleeve Material	Resin Matrix	Inner Diameter (mm)	Outer Diameter (mm)	Inner Material	Storage
A	Woven	BisGMA	1.5	2	Gradia	Dry or aged
B		PMMA				
C		BisGMA	1.2			
D		PMMA				
E		BisGMA	1.5		MI-post	
F					E-glass	
G	Sleeve fiber net	BisGMA	1.5		Gradia	
H		PMMA				
I (control)	i-TFC sleeve				i-TFC post	

## Data Availability

The data presented in this study are available on request from the corresponding author.
